# Cytokinetic Failure-induced Tetraploidy Develops into Aneuploidy, Triggering Skin Aging in Phosphovimentin-deficient Mice*

**DOI:** 10.1074/jbc.M114.633891

**Published:** 2015-04-06

**Authors:** Hiroki Tanaka, Hidemasa Goto, Akihito Inoko, Hiroyuki Makihara, Atsushi Enomoto, Katsuhisa Horimoto, Makoto Matsuyama, Kenichi Kurita, Ichiro Izawa, Masaki Inagaki

**Affiliations:** From the ‡ Division of Biochemistry, Aichi Cancer Center Research Institute, Nagoya 464-8681,; the Departments of § Cellular Oncology and; ‖ Pathology, Nagoya University Graduate School of Medicine, Nagoya 466-8550,; the ¶ Department of Oral and Maxillofacial Surgery, School of Dentistry, Aichi Gakuin University, Nagoya 466-8550, and; the ** Molecular Profiling Research Center for Drug Discovery, National Institute of Advanced Industrial Science and Technology, Tokyo 135-0064, Japan

**Keywords:** aging, cytokinesis, intermediate filament, phosphorylation, skin, aneuploidy, tetraploidy

## Abstract

Tetraploidy, a state in which cells have doubled chromosomal sets, is observed in ∼20% of solid tumors and is considered to frequently precede aneuploidy in carcinogenesis. Tetraploidy is also detected during terminal differentiation and represents a hallmark of aging. Most tetraploid cultured cells are arrested by p53 stabilization. However, the fate of tetraploid cells *in vivo* remains largely unknown. Here, we analyze the ability to repair wounds in the skin of phosphovimentin-deficient (*VIM^SA/SA^*) mice. Early into wound healing, subcutaneous fibroblasts failed to undergo cytokinesis, resulting in binucleate tetraploidy. Accordingly, the mRNA level of p21 (a p53-responsive gene) was elevated in a *VIM^SA/SA^*-specific manner. Disappearance of tetraploidy coincided with an increase in aneuploidy. Thereafter, senescence-related markers were significantly elevated in *VIM^SA/SA^* mice. Because our tetraploidy-prone mouse model also exhibited subcutaneous fat loss at the age of 14 months, another premature aging phenotype, our data suggest that following cytokinetic failure, a subset of tetraploid cells enters a new cell cycle and develops into aneuploid cells *in vivo*, which promote premature aging.

## Teaser

**Results:** Tetraploid skin fibroblasts enter a new cell cycle and develop into aneuploid fibroblasts in phosphovimentin-deficient mice.

**Conclusion:** These mice exhibited aging phenotypes in the skin.

**Significance:** Our data suggest a possible causal relationship between tetraploidy and premature aging.

## Introduction

Although eukaryotes possess diploid chromosome sets, some mammalian cells contain four (tetraploid) chromosome sets in certain physiological and pathological settings. Tetraploidy is observed during the process of differentiation in liver tissue where it is considered an adaptation to meet the needs for high metabolic activity (1–3). Furthermore, the proportion of polyploidy (including tetraploidy) is elevated during the aging process in several tissues (4–6). Approximately 20% of solid tumors exhibit tetraploid and near-tetraploid karyotypes (7). A recent study using 11 types of cancers suggests that 37% of cancers have passed through a tetraploid stage (a whole-genome doubling event) during their development (8). Tetraploid cells with genetic alterations of cancer-related gene(s) exhibit chromosomal instability and can form tumors in nude mice (9, 10). Thus, accumulating evidence suggests a model in which tetraploid cells emerge early in carcinogenesis and develop into aneuploid cells with aberrant chromosome numbers (11–15).

Tetraploid cells can be generated by a variety of different insults, but mitotic slippage and cytokinetic failure are considered as two major routes for tetraploidization *in vivo* (7, 12, 15). Several studies using cultured cells have demonstrated that the proliferation of tetraploid cells is limited largely by the tumor suppressor protein p53; the resulting cell cycle arrest is referred to as “post-mitotic checkpoint” (16) or “tetraploidy checkpoint” (17–19). A recent report has demonstrated that tetraploidy due to cytokinetic failure activates the Hippo signaling pathway, which results in the stabilization of p53 (20). However, the behavior of tetraploid cells *in vivo* remains largely unknown, partly because no tetraploidy-prone mice were available.

The widely expressed intermediate filament (IF)^2^ protein vimentin is found in mesenchymal cells and the eye lens (21–25). Vimentin is phosphorylated predominantly in mitosis (26), which is regulated by several mitotic kinases such as Aurora-B (27), Cdk1 (28–30), Polo-like kinase 1 (Plk1) (31), and Rho kinase (32). In cultured cells, mitotic vimentin phosphorylation is of great importance for the completion of cytokinesis (31). Interference with vimentin phosphorylation retains an IF bridge-like structure (IF-bridge) (33) connecting the two daughter cells during cytokinesis, the end of mitosis (27, 34). Knock-in mice expressing only vimentin mutated from Ser to Ala at Aurora-B, Cdk1, Plk1, and Rho kinase sites (*VIM^SA/SA^* mice) developed binucleation (tetraploidy) and aneuploidy in lens epithelial cells and exhibited a lens cataract, a prominent progeroid phenotype (35, 36). However, whether binucleate tetraploidy precedes aneuploidy or whether these events occur independently in *VIM^SA/SA^* mice remains unknown.

In this study, we address this by analyzing dorsal skin wound healing in *VIM^SA/SA^* mice. In response to skin injury, vimentin expression was elevated at wound areas of subcutaneous fibroblasts in a genotype-independent manner. During the acute phase of wound healing when vimentin expression was relatively high, IF-bridge formation, binucleation (tetraploidy), and extra-centrosome formation were observed specifically in *VIM^SA/SA^* fibroblasts. These cellular structures disappeared with decreased vimentin expression, leading to increased numbers of aneuploid fibroblasts. Subsequently, *VIM^SA/SA^* fibroblasts exhibited a significant elevation of major senescence-related markers. These abnormalities resulted in impaired wound healing, one of the premature aging phenotypes.

## Experimental Procedures

### 

#### 

##### Mice

Knock-in mice carrying vimentin mutations were generated and characterized as reported earlier (36). These mice were backcrossed onto and maintained on the C57BL/6c background in a specific pathogen-free facility. Animal experiment protocols were approved by the Animal Ethics and Animal Care Committees at the Aichi Cancer Center.

##### Skin Wound Healing Assay

Full thickness excisional wounds were created on the middle dorsal region of mice aged 3 months with a sterile 8-mm diameter biopsy punch (Disposal BIOPSY PUNCH inner diameter of 8 mm; Kai Industries Co., Ltd., Tokyo, Japan). The wounds were left open, and the animals were housed in individual cages. Each wound site was digitally photographed every other day after injury, and the extent of wound closure was quantified by measuring the area of wound remaining open using the image processing software (Adobe Photoshop, San Jose, CA). Wound tissue and surrounding wound margin skin were harvested from mice at indicated days post-wounding and fixed in 4% paraformaldehyde in PBS prior to paraffin embedding and sectioning at 5 μm.

##### Murine Dissection, Slice Preparation, Histology, Immunohistochemistry, and Immunofluorescence

3- or 14-month-old mice were sacrificed under general anesthesia, perfused with 10% neutral buffered formalin, postfixed in the same fixative overnight, embedded in paraffin, and cut into 5-μm sections. Conventional hematoxylin and eosin (H&E) staining, immunohistochemistry, or immunofluorescence was performed as described previously (36). Picro Sirius Red staining was performed as below. Paraformaldehyde-fixed tissue sections were incubated at 60 °C for 45 min. These were stained with 0.1% (w/v) Sirius red (Sigma) and 0.1% (w/v) Fast Green (Sigma) dissolved in saturated aqueous solution of picric acid (Sigma) for 5–10 min. Then, they were deparaffinized and stained with 0.1% (w/v) Sirius red dissolved in picric solution for 60 min. After staining, they were washed with acidified water (1% (v/v) acetic acid water) and distilled water, respectively. These were dehydrated, mounted in xylene, and finally sealed with Malinol (Muto Pure Chemicals Co., Ltd., Tokyo, Japan). For the analysis of heart size, maximum transverse sections were prepared, followed by the measurement of the diameter of the left ventricles. For the measurement of the thickness of the aortic media, the abdominal aorta was resected *en bloc* with the surrounding connective tissues, followed by fixation, sectioning, and microscopic examination. The weight of the mesenteric fat was measured by resecting the mesentery from fixed gut tracts.

##### Antibodies

Rabbit polyclonal anti-β-gal and mouse monoclonal anti-centrin (clone 20H-5) antibodies were purchased from Abcam (Cambridge, MA) or Millipore (Temecula, CA), respectively. Rabbit polyclonal anti-keratin 1, 6, and 14 antibodies were kindly provided by Dr. T. Magin (University Leipzig, Leipzig, Germany). Other primary and secondary antibodies were used as described elsewhere (36, 37).

##### FISH and Quantitative Real Time RT-PCR

FISH and quantitative real time RT-PCR were performed as described previously (36).

##### Primary Cell Culture

Primary fibroblasts or mouse embryonic fibroblasts (MEFs) were established from neonatal mouse skin or mouse whole embryo, respectively. These fibroblasts were cultured in DMEM with 10% FBS and supplemented with 100 mg/ml streptomycin and 100 units/ml penicillin. Interphase or mitotic fibroblasts were prepared as follows. The cells were incubated with 100 μm monastrol (Biomol International, Plymouth Meeting, PA) for 6 h to synchronize the cells at prometaphase (Fig. 7, *D* and *F*). In Fig. 7*D*, treated cells were washed with PBS three times and then incubated with 20 μm MG132 (Merck, Darmstadt, Germany) for 90 min to block the exit from metaphase. These mitotic cells were collected by mechanical shake off (Fig. 7, *D* and *F*), and adherent cells were used as interphase cells (Fig. 7*D*).

##### Fractionation Assay

Mitotic MEFs were incubated at 4 °C for 30 min in the buffer containing 50 mm Tris-Cl (pH 7.5), 50 mm NaCl, 50 mm sodium pyrophosphate, 50 mm NaF, 50 mm sodium β-glycerophosphate, 1 mm Na_3_VO_4_, 2 mm EDTA, and 1% Triton X-100. After the incubation, these samples were centrifuged at 10,000 × *g* for 10 min and then separated into the supernatant and pellet fractions. Each fraction was subjected to immunoblotting with rabbit monoclonal anti-vimentin (clone EPR3776, Abcam), anti-histone-H3 (HH3; clone D1H2), or HRP-conjugated anti-GAPDH (clone 14C10, Cell Signaling Technology, Beverly, MA) antibodies.

##### Immunoprecipitation

MEFs were lysed in the buffer containing 50 mm Tris-Cl (pH 7.5), 150 mm NaCl, 50 mm sodium pyrophosphate, 50 mm NaF, 50 mm sodium β-glycerophosphate, 1 mm Na_3_VO_4_, 2 mm EDTA, 0.5% deoxycholate, 0.1% SDS, and 1% Nonidet P-40. After the centrifugation (17,400 × *g*), each supernatant was incubated with 2 μl of goat anti-vimentin (antisera) (34) for 30 min, followed by an additional 30-min incubation with 10 μl of protein G-Sepharose beads (Life Technologies, Inc.). All the above procedures were performed at 4 °C. Each immunoprecipitate was immunoblotted with rabbit anti-vimentin or mouse anti-O-GlcNAc (clone CTD110.6, Cell Signaling Technology) monoclonal antibodies.

##### Western Blotting after Mn^2+^-Phos-Tag SDS-PAGE

Mn^2+^-Phos-tag SDS-PAGE was performed as described previously (38), with a slight modification. Each cell lysate was subjected to Mn^2+^-Phos-tag SDS-PAGE (8% polyacrylamide gel, including 20 μm Phos-tag acrylamide (Wako Pure Chemical, Osaka, Japan) and 40 μm MnCl_2_) and then analyzed by Western blotting.

##### Statistical Analyses

Antibody signals in a digital image of Figs. 5*C* and 6*B* were calculated as described previously (39). All data were shown as mean ± S.E. All *p* values (*, *p* < 0.05; **, *p* < 0.01; ***, *p* < 0.001) were determined by two-tailed Student's *t* test (Graph Pad software), compared with wild-type (*VIM^WT/WT^*) mice.

## Results

### 

#### 

##### Mitotic Vimentin Phosphorylation-deficient (VIM^SA/SA^) Mice Exhibit Subcutaneous Fat Loss at Age 14 Months

**FIGURE 1.**
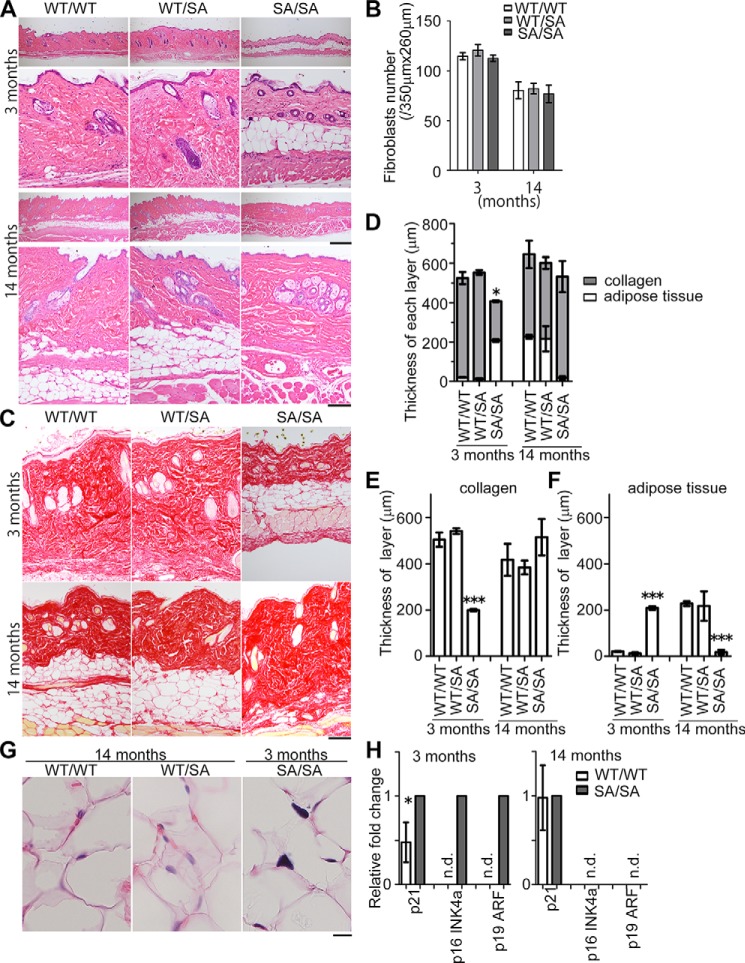
**Skin tissue of mitotic vimentin phosphorylation-deficient mice (*VIM^SA/SA^*).** *A* and *B*, each photograph shows H&E staining of dorsal skin derived from 3- or 14-month-old mice (*A*). We calculated the average number of subcutaneous fibroblasts in the affected area at the indicated days, using more than 100 H&E sections per wound (*B*). Data represent mean ± S.E. of subcutaneous fibroblasts per 350 × 260-μm wound area (*n* = 6 or 3 mice per genotype at age 3 or 14 months, respectively; *B*). *C–F,* each photograph shows Picro Sirius Red staining of dorsal skin. Collagen fibers were stained *red* (*C*). We calculated the average thickness of subcutaneous collagen or fat (adipose) layer in dorsal skin, using more than 20 sections per mouse; data represent mean ± S.E. of three independent experiments (*, *p* < 0.05, two-tailed *t* test; *D–F*). *G*, magnified H&E images show adipocytes in dorsal skin. *H*, amounts of mRNA of the indicated genes at dorsal skin were quantified using real time RT-PCR, normalized to the amount of GAPDH mRNA, and presented as fold of *VIM^SA/SA^* mice. Data represent mean ± S.E. of four independent experiments. *Scale bars* = 500 μm (*A*, *upper panels*), 200 μm (*A*, *lower panels* and *C*), and 10 μm (*G*). *n.d.,* no detected signals. ***, *p* < 0.001).

Because vimentin is preferentially expressed in mesenchymal cells, including subcutaneous fibroblasts (21–25), we first compared differences in subcutaneous tissue of wild-type (*VIM^WT/WT^*), heterozygotic (*VIM^WT/SA^*), and homozygotic (*VIM^SA/SA^*) mice. There were marginal changes in cell density of subcutaneous fibroblasts among three genotypes at the age 3 or 14 months (Fig. 1, *A* and *B*), but the thickness of the dermis (especially of the dermal collagen layer) was significantly decreased in *VIM^SA/SA^* mice at the age 3 months (Fig. 1, *A* and *C–E*). At the same time, the subcutaneous fat (adipose) layer was increased in 3-month-old *VIM^SA/SA^* mice, whereas no apparent fat layer was detected in *VIM^WT/WT^* and *VIM^WT/SA^* mice at the same age (Fig. 1, *A*, *C*, *D*, and *F*). By the age of 14 months, the subcutaneous fat layer was almost absent in *VIM^SA/SA^* mice (Fig. 1, *A*, *C*, *D*, and *F*). At the same time, the subcutaneous fat layer in *VIM^WT/WT^* and *VIM^WT/SA^* mice increased with age (Fig. 1, *A*, *C*, *D*, and *F*). In *VIM^SA/SA^* mice, the nuclei of subcutaneous adipose cells appeared to increase in size and in staining intensity (Fig. 1*G*), suggesting that cytokinetic failure might occur in these adipocytes. Real time PCR analyses using skin tissue revealed that mRNA expression of p21, p16^INK4a^, and p19^ARF^ (senescence-related genes) (40–43) was significantly elevated in 3-month-old *VIM^SA/SA^* mice (Fig. 1*H*). Because subcutaneous fat loss was observed at the age of 12 months but not of 2 months in aneuploidy-prone, *BubR1^H/H^* progeria mice (44), these results suggested that subcutaneous fat loss at the age of 14 months likely represents a major progeroid feature in *VIM^SA/SA^* mice.

##### VIM^SA/SA^ Mice Exhibit No Apparent Phenotypes in Liver, Kidney, Mesentery, Heart, or Aorta

**FIGURE 2.**
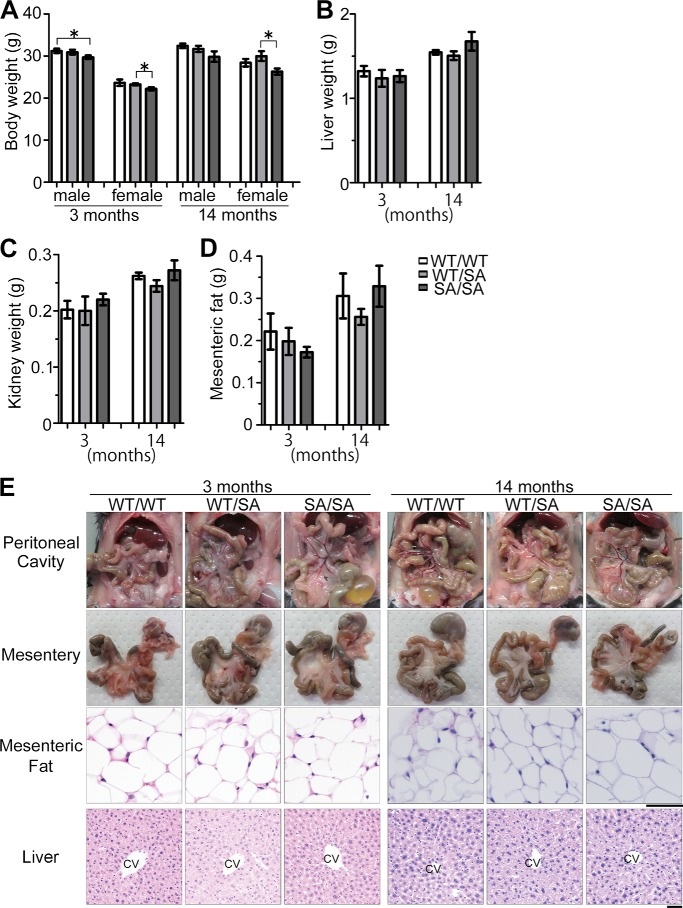
***VIM^SA/SA^* mice exhibit weight loss but not any phenotypes in liver, kidney, and mesentery.** *A–D*, body weight of male or female mice (*n* = 10) or each tissue weight of male mice (*n* = 5) was measured. *E*, each photograph shows gross appearance of abdominal organs and mesentery. Liver and mesenteric fat tissues were also stained with H&E. *CV* indicates central vein in the liver. *Scale bars,* 50 μm. *, *p* < 0.05.

*VIM^SA/SA^* mice also exhibited a tendency to lose body weight (Fig. 2*A*). However, there were no significant differences in organ size, such as liver (Fig. 2*B*) or kidney (Fig. 2*C*). We then evaluated the visceral fat. In the mesentery, there are only marginal changes in the weight (Fig. 2*D*) and morphology (Fig. 2*E*) of mesenteric fat tissue among three genotypes. In addition, no fatty liver was developed in all three types of mice (Fig. 2*E*). Therefore, the abnormalities of fat distribution in *VIM^SA/SA^* mice are likely restricted to subcutaneous tissue.

**FIGURE 3.**
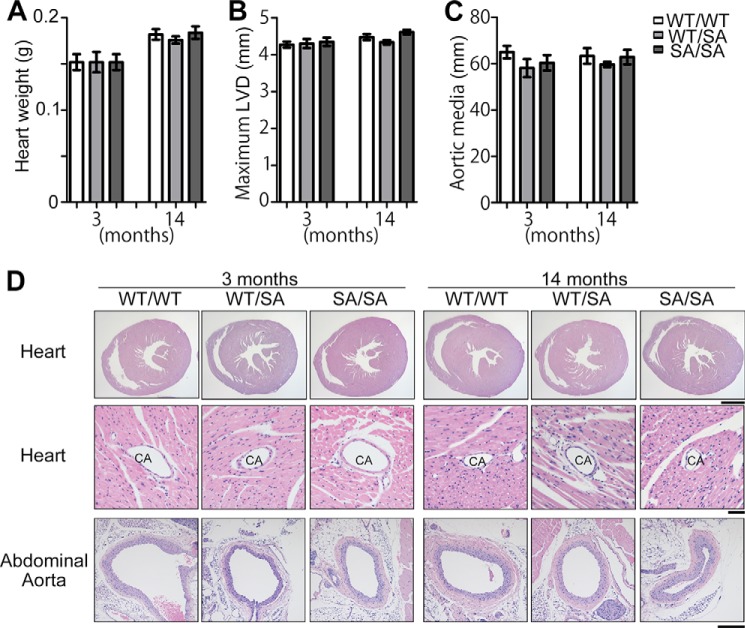
***VIM^SA/SA^* mice exhibit no abnormalities in the cardiovascular system.** *A–C*, heart weight, maximum left ventricular diameter (*LVD*), or the thickness of abdominal aortic media was measured using each group of male mice (*n* = 5). *D*, hearts and abdominal aortic media were stained with H&E. *CA* indicates coronary artery in the heart. *Scale bars,* 1,000 μm (*D*, *upper panels*), 50 μm (*D*, *middle panels*), and 200 μm (*D*, *lower panels*).

Judged by heart weight (Fig. 3*A*), maximum left ventricular diameter (Fig. 3*B, LVD*), the thickness of abdominal aortic media (Fig. 3*C*), and H&E staining of heart (including coronary arteries) and abdominal aorta (Fig. 3*D*), *VIM^SA/SA^* mice exhibited no apparent abnormalities or diseases in cardiovascular system.

##### VIM^SA/SA^ Mice Show a Significant Delay in Wound Repair Involving Dysfunctional Subcutaneous Fibroblasts

**FIGURE 4.**
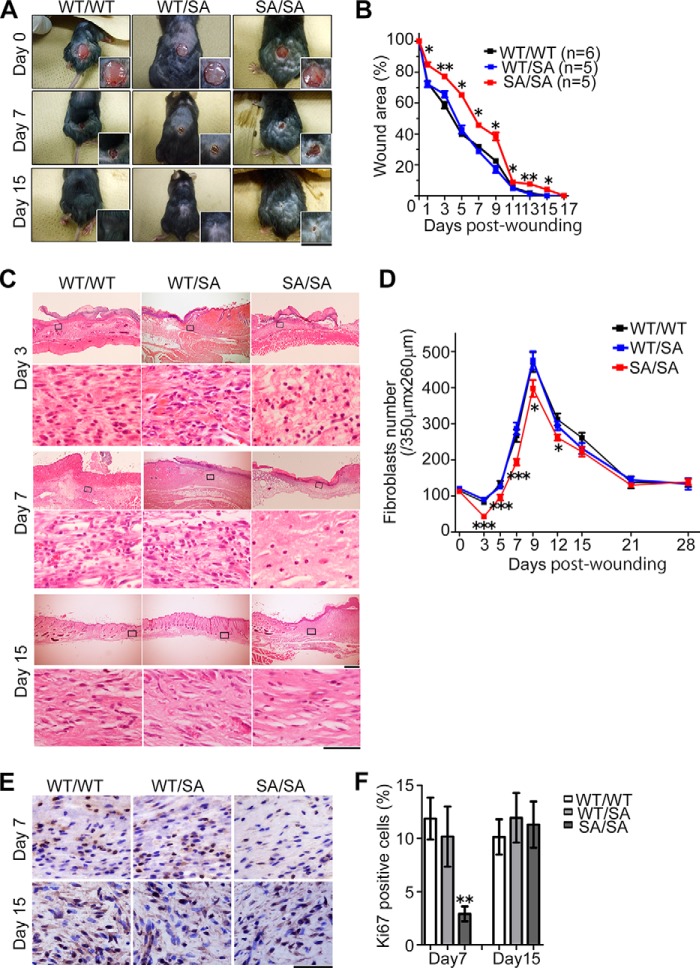
**Delayed wound repair in *VIM^SA/SA^* mice.** *A* and *B*, time course of wound closure process after the wounding of dorsal skin. The *graph* shows the percentage of remaining affected area at the indicated days after skin injury; the area at day 0 was set at 100% (*B*). *C* and *D*, each photograph shows H&E staining of dorsal skin at the indicated days after the injury (*C*). We calculated the average number of subcutaneous fibroblasts in the affected area as described in the legend of Fig. 1 (*D*). *E* and *F*, wound area of subcutaneous tissue was stained with anti-Ki67 at 7 or 15 days after the injury (*E*). We calculated the average proportion of Ki67-positive fibroblasts in the affected area at the indicated days, using more than 20 sections for each wound; data represent mean ± S.E. of six independent experiments (*F*). *Scale bars,* 1,000 μm (*A*), 500 μm (*C*, low magnification), and 50 μm (*C*, high magnification, and *E*). *, *p* < 0.05; **, *p* < 0.01; ***, *p* < 0.001.

**FIGURE 5.**
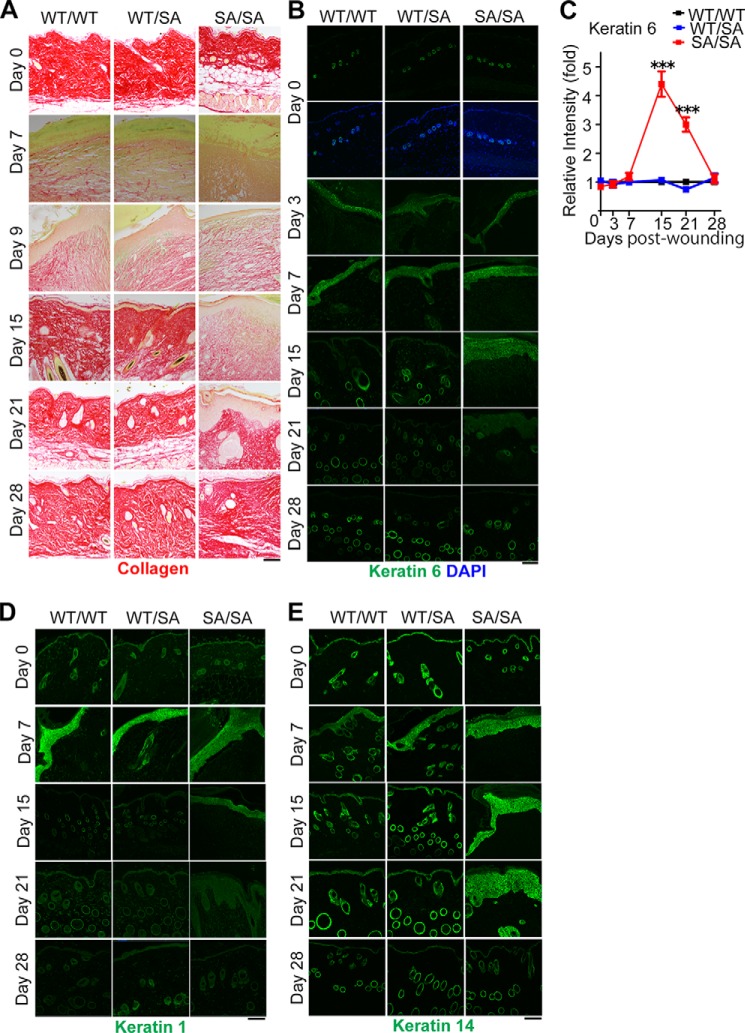
**Dysfunction of skin fibroblasts and epithelial cells during wound repair in the *VIM^SA/SA^* mice.** *A–E*, each photograph shows Picro Sirius Red (*A*), anti-keratin 6 (*B*), anti-keratin 1 (*D*), or anti-keratin 14 (*E*) staining of dorsal skin at the center of wound region. We calculated the relative intensity of anti-keratin 6, using more than 20 sections per mouse (*C*). Data are presented as fold of *VIM^SA/SA^* mice at each day after the injury and represent mean ± S.E. of six independent experiments. ***, *p* < 0.001. *Scale bars,* 200 μm.

Because impaired wound healing was also identified as one of the age-related pathologies in other progeroid mouse models (40, 41, 44), we next analyzed the ability to repair wounds in 3-month-old *VIM^SA/SA^* mice. Compared with *VIM^WT/WT^* or *VIM^WT/SA^* mice, *VIM^SA/SA^* mice showed a significant delay in wound closure after the injury of dorsal skin (Fig. 4, *A* and *B*). In *VIM^SA/SA^* mice, the number of subcutaneous fibroblasts at affected areas was significantly reduced from 3 to 12 days after skin injury (Fig. 4, *C* and *D*). Judged by anti-Ki67 staining, proliferation in fibroblasts was significantly decreased in subcutaneous tissue of *VIM^SA/SA^* mice at 7 days; proliferation rates returned to normal by 15 days after skin injury (Fig. 4, *E* and *F*). Subcutaneous collagen deposition was severely impaired at the affected area in *VIM^SA/SA^* mice (Fig. 5*A*), suggesting the dysfunction of subcutaneous fibroblasts in *VIM^SA/SA^* mice. To evaluate skin re-epithelialization during wound healing, we analyzed the expression of keratins, epithelium-specific IF proteins. 3 days after the injury, keratin 6 (one isoform of type II basic keratins) was highly expressed in all genotypes (Fig. 5, *B* and *C*); this phenomenon was consistent with the previous observation that keratin 6 expression was up-regulated after skin injury (45). Keratin 6 expression returned to normal in *VIM^WT/WT^* or *VIM^WT/SA^* mice by 15 days after the injury, but high expression levels were sustained in *VIM^SA/SA^* mice, suggesting disturbance of keratinocyte differentiation (Fig. 5, *B* and *C*) (46). A similar tendency was observed in the case of keratin 1 (differentiation-specific type II basic keratin; Fig. 5*D*) or keratin 14 (type I acidic keratin expressed in the basal layer of the epidermis; Fig. 5*E*). Immunostaining with anti-keratin antibodies also revealed prolonged, transient hyperkeratosis during the repair process in *VIM^SA/SA^* mice (Fig. 5, *A*, *B*, *D*, and *E*). Therefore, dysfunction of subcutaneous fibroblasts likely affects re-epithelialization steps during wound repair in *VIM^SA/SA^* mice.

##### Association of Phosphorylation-deficient Vimentin with Tetraploid Fibroblasts Showing Extra Centrosomes during Acute Phase of Wound Repair

**FIGURE 6.**
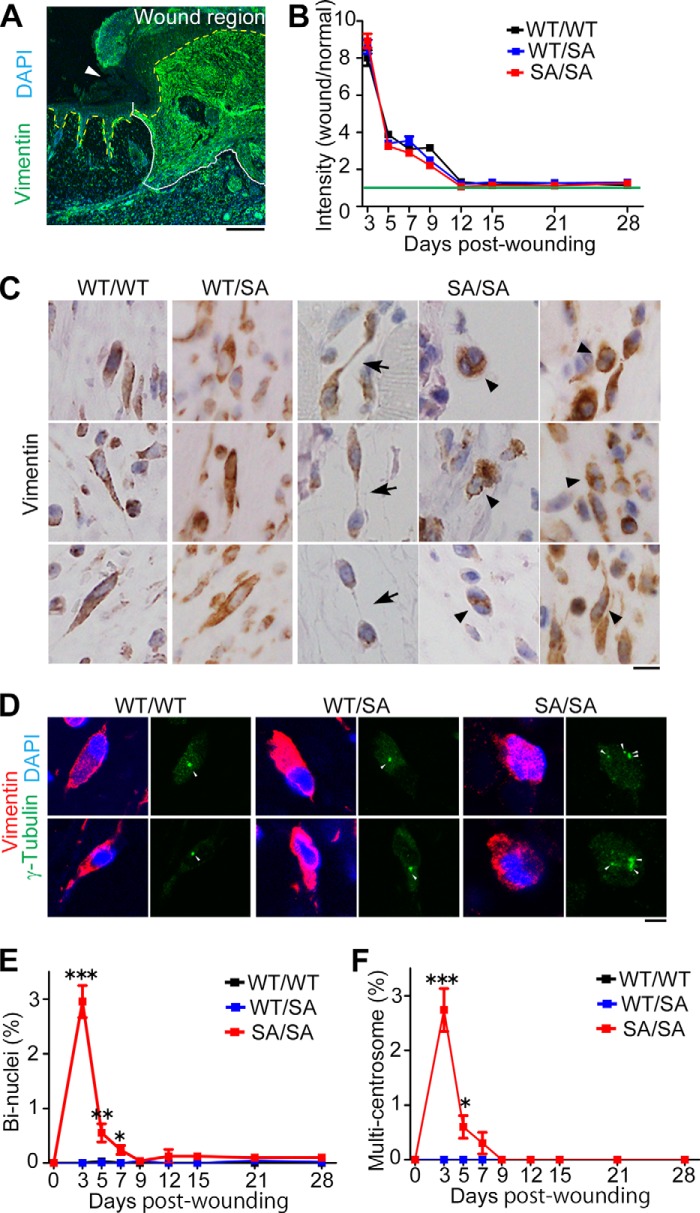
**Formation of multinuclei, IF-bridge, and extra centrosomes in *VIM^SA/SA^* fibroblasts at early stage of wound healing.** *A* and *B*, histological sections after wounding were stained with anti-vimentin (*green*) and DAPI (*blue*). An immunofluorescent micrograph of the *VIM^WT/WT^* mouse at day 7 after wounding is shown as a representative sample (*A*). *White dot* and *yellow dashed lines* are indicated as the borders between wound and unaffected (normal) areas and between epidermis and dermis, respectively (*A*). The position of wound edge is also marked by *arrowhead* (*A*). We calculated the ratio of anti-vimentin intensity at wound areas to at neighboring unaffected (normal) areas, using 10 sections per each wound (*B*). Data represent mean ± S.E. of three independent experiments (*B*). A 1:1 ratio is indicated as a *green line* in the graph (*B*). *C–F*, immunohistochemical (*C*) or immunofluorescent (*D*) images of affected areas at day 3 after wounding. *Arrows* or *arrowheads* in *C* indicate fibroblasts with IF-bridge (connecting two daughter cells) or two nuclei, respectively. *Arrowheads* in *D* indicate γ-tubulin spots in fibroblasts. We calculated the percentage of subcutaneous fibroblasts with bi-nuclei (*E*) or more than three γ-tubulin spots (centrosomes; *F*) at the indicated days, using more than 10 sections per each wound. Data represent mean ± S.E. of three independent experiments (*E* and *F*). *Scale bars,* 500 μm (*A*), 10 μm (*C*), and 5 μm (*D*). *, *p* < 0.05; **, *p* < 0.01; ***, *p* < 0.001.

Because tissue injury increased vimentin expression in a TGFβ1-dependent manner (47) required for wound repair (48, 49), we analyzed the relationship between vimentin expression and fibroblastic anomalies during wound repair. Vimentin expression was elevated >8-fold in wound areas at day 3, but it returned to the level indistinguishable from the neighboring unaffected areas around 12 days after injury. There were, if at all, marginal changes in vimentin expression among the three genotypes (Fig. 6, *A* and *B*). An “IF-bridge” phenotype (Fig. 6*C*, *arrows*) and binucleation (Fig. 6*C*, *arrowheads*) were detected in a subset of subcutaneous fibroblasts of *VIM^SA/SA^* mice, although such abnormal structures were not observed in the littermate controls. We also noted fibroblasts with more than three γ-tubulin spots representing centrosomes in a *VIM^SA/SA^*-specific manner (Fig. 6*D*); the fibroblasts with the above abnormalities appeared randomly rather than exhibiting specific tissue localization (data not shown). The percentage of binucleation (Fig. 6*E*) or extra centrosome formation (Fig. 6*F*) peaked at day 3 and rapidly decreased thereafter. In contrast to a normal cell cycle, the existence of fibroblasts with more than three centrosomes indicates that binuclear fibroblasts with two centrosomes undergo additional cell cycles rather than arrest at a certain cell cycle stage.

**FIGURE 7.**
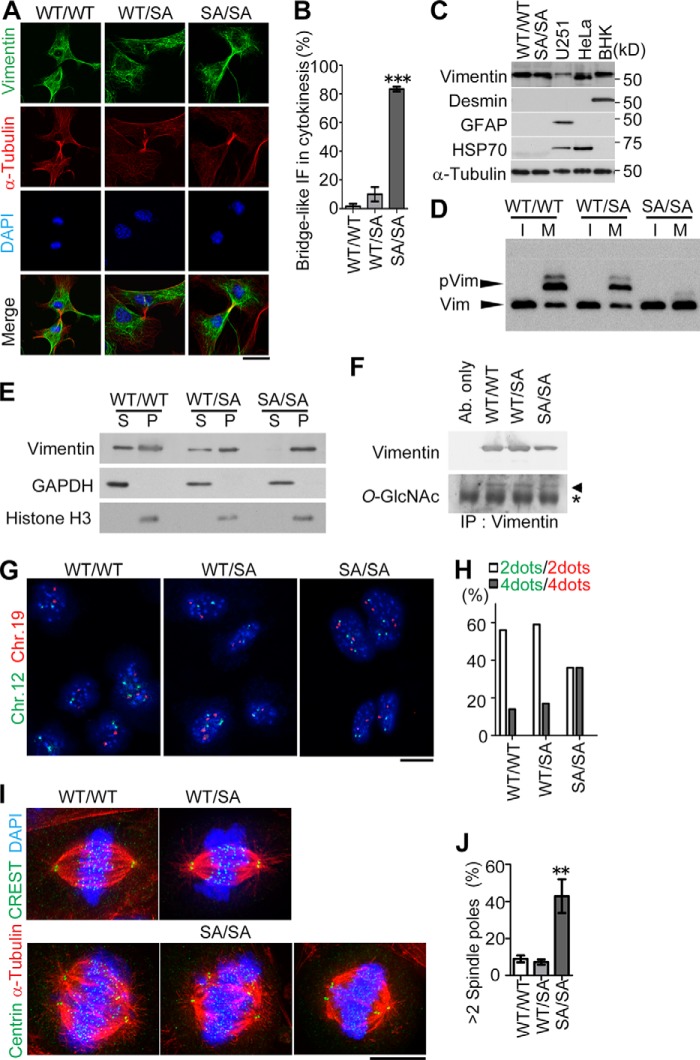
**Detailed analysis of primary cultured cells derived from neonatal mouse dorsal skin.** *A–J*, primary fibroblasts from mouse neonatal skin (*A–D* and *G–J*) or mouse embryonic fibroblasts (*E* and *F*) were subjected to immunostaining (*A*, *B*, *I*, and *J*), immunoblotting (*C–F*), or FISH (*G* and *H*). *C*, U251 (human glioma), HeLa (human cervical carcinoma), or baby hamster kidney (*BHK*) cells were used as positive controls for the detection of glial fibrillary acidic protein, HSP70, or desmin, respectively. *D*, interphase (*I*) or mitotic (*M*) cell lysates were prepared as described under “Experimental Procedures” and then subjected to Mn^2+^-Phos-tag SDS-PAGE followed by immunoblotting. Phosphorylated vimentin (*pVim*) migrated slower than vimentin without phosphorylation (*Vim*) due to the interaction of a phosphate group with Mn^2+^-Phos-tag-modified polyacrylamide. *E*, the supernatant (*S*) and pellet (*P*) fractions of mitotic MEFs were prepared as described under “Experimental Procedures.” The amount of GAPDH or histone H3 (*HH3*) was also monitored for the evaluation of fractionation. *F*, vimentin immunoprecipitated (*IP*) from MEFs was subjected to the immunoblotting. The same experimental procedures were also performed without any cells as a negative control (*Ab. only*). An *asterisk* or *arrowhead* indicates the position of IgG heavy chain or vimentin, respectively. *G*, *green* or *red color* represents the spots of mouse chromosome (*Chr.*) 12 or 19, respectively. The percentage of cells with IF-bridge (*B*) or over two spindle poles (*J*) was calculated using at least 20 cells per experiment. Data represent mean ± S.E. of three independent experiments (*B* and *J*). *Scale bars,* 10 μm (*A* and *G*) and 5 μm (*I*). *, *p* < 0.05; **, *p* < 0.01; ***, *p* < 0.001.

To analyze the molecular mechanism more precisely, we established primary fibroblasts from neonatal mouse skin (Fig. 7, *A–D* and *G–J*) or MEFs from mouse embryos (Fig. 7, *E* and *F*). Compared with the *in vivo* setting, vimentin expression was significantly elevated in cultured cells (not depicted; also see Fig. 7*C*) as reported previously (50). The majority of *VIM^SA/SA^*-derived primary culture fibroblasts exhibited IF-bridge phenotypes in cytokinesis (Fig. 7, *A* and *B*), similar to our previous observations following transient expression of the SA mutant in T24 cells (27, 31, 34). In primary fibroblasts from neonatal skin, we observed neither redundant protein expression of desmin and glial fibrillary acidic protein (with which vimentin can form heteropolymeric filaments) (51) nor heat shock protein 70 (HSP70) expression, which is elevated in lens fibers accumulating vimentin aggregates (Fig. 7*C*) (52). To examine whether or not vimentin was phosphorylated in mitotic primary fibroblasts, we performed Mn^2+^-Phos-tag SDS-PAGE (53, 54) followed by Western blotting. Because of the interaction of a phosphate group with Mn^2+^-Phos-tag-modified polyacrylamide, phosphorylated vimentin (Fig. 7*D*, *pVim*) migrated slower than vimentin without phosphorylation (Fig. 7*D*, *Vim*). A proportion of vimentin was phosphorylated specifically during mitosis in *VIM^WT/WT^*- or *VIM^WT/SA^*-derived primary culture fibroblasts. However, no bands corresponding to phosphorylated vimentin (pVim) were detected in *VIM^SA/SA^*-derived mitotic fibroblasts, suggesting that no compensatory phosphorylation occurred on SA mutant in mitosis. The fractionation assay revealed that vimentin in mitotic *VIM^SA/SA^* MEFs was detected predominantly in the pellet (*P*) fraction (Fig. 7*E*). However, vimentin in mitotic *VIM^WT/WT^* or *VIM^WT/SA^* MEFs was to some extent recognized in the supernatant (*S*) fraction (Fig. 7*E*). Because not only phosphate but also *N*-acetyl-d-glucosamine (GlcNAc) are post-translationally linked to Ser/Thr residues on vimentin (55), we purified vimentin as an anti-vimentin immunoprecipitate from each type of MEF and then immunoblotted it with anti-*O*-GlcNAc. Because we observed only marginal changes in the intensity of anti-*O*-GlcNAc (Fig. 7*F*), it is unlikely that *O*-linked glycosylation may be affected by vimentin mutations at mitotic phosphorylation sites (Ser residues) to Ala (SA mutations). Therefore, all these data suggested that vimentin filament solubility is increased by mitotic vimentin phosphorylation, the impairment of which results in IF-bridge formation between two daughter cells.

Nuclei with two FISH signals per diploid chromosome (implying diploid) were detected less frequently in *VIM^SA/SA^* primary fibroblasts from neonatal skin (Fig. 7, *G* and *H*). Instead, the population of nuclei with four FISH signals (implying tetraploid) increased in *VIM^SA/SA^* cells (Fig. 7, *G* and *H*). In addition, >2 spindle poles were much more frequently formed in mitotic fibroblasts derived from *VIM^SA/SA^* than from *VIM^WT/WT^* or *VIM^WT/SA^* (Fig. 7, *I* and *J*). These results suggest that most primary fibroblasts derived from *VIM^SA/SA^* mice are competent to enter a new cell cycle after they become tetraploid via cytokinetic failure by compromised mitotic vimentin phosphorylation.

##### Tetraploid Fibroblasts with Duplicated Centrosomes Develop into Aneuploid Cells, Exhibiting Cellular Senescence during Late Stages of Wound Repair

**FIGURE 8.**
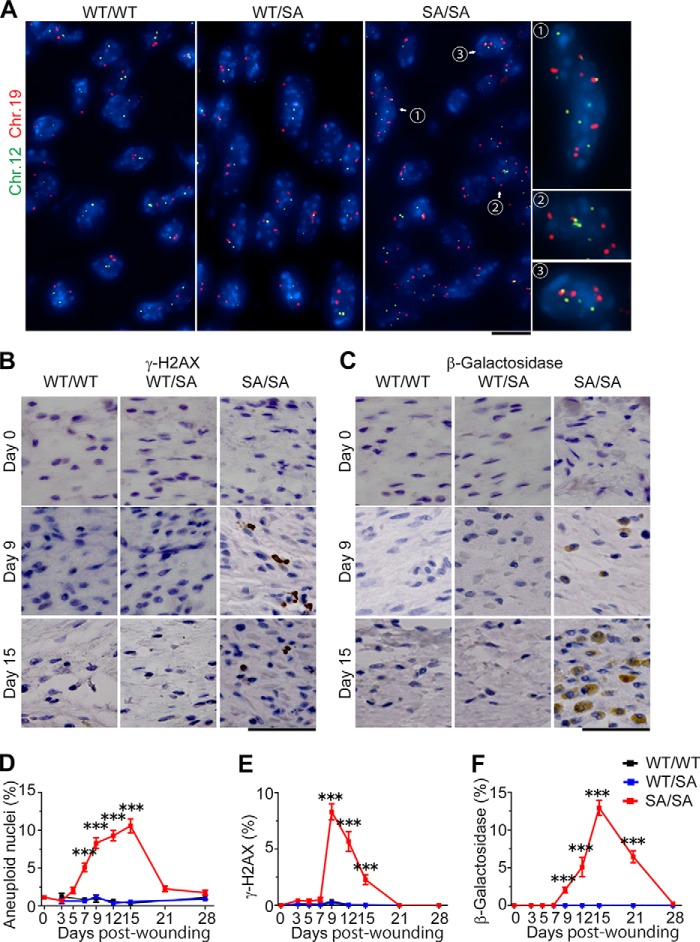
**Emergence of aneuploid and senescent fibroblasts at later stage of wound healing in *VIM^SA/SA^* mice.** *A* and *D*, photos show the FISH analyses of fibroblastic nuclei at affected areas at the indicated days after skin injury. Photographs of mice with each genotype at day 15 after wounding are shown as representative samples (*A*). The *graph* shows the percentage of aneuploid fibroblasts (*n* = 10 sections per genotype; *D*). *B*, *C*, *E*, and *F*, histological sections at 0, 9, or 15 days after wounding were stained with anti-γ-H2AX (*B*) or anti-β-galactosidase (β-*galactoside*; *C*). We calculated the percentage of γ-H2AX (*E*)- or β-gal (*F*)-positive fibroblasts at affected areas, using at least 10 sections per wound. Data represent mean ± S.E. of three independent experiments (*E* and *F*). *Scale bars,* 10 μm (*A*) and 50 μm (*B* and *C*). ***, *p* < 0.001.

**FIGURE 9.**
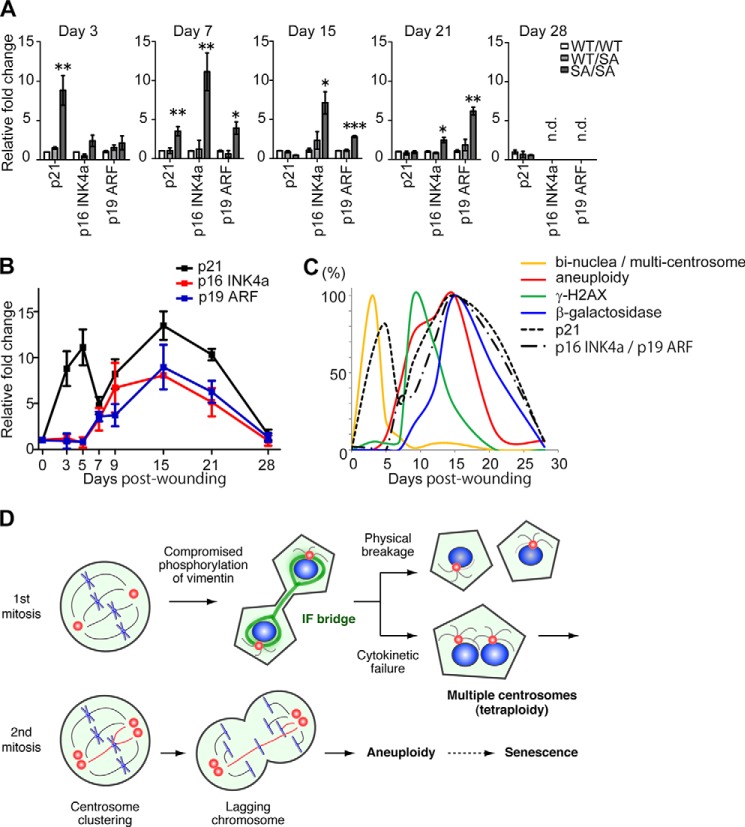
**Elevation of senescent markers in *VIM^SA/SA^* mice during wound healing.** *A* and *B*, amounts of mRNA of the indicated genes at affected areas were quantified using real time RT-PCR, as described in the legend of Fig. 1F. The data were presented as fold of VIM*^WT/WT^* mice at the indicated day after the injury to compare the difference among genotypes (*A*) or as fold of *VIM^SA/SA^* mice before the injury (at day 0) to analyze the time course of mRNA expression in *VIM^SA/SA^* mice (*B*). *C,* judged from the data in Figs. 6, *E* and *F*, 8, *D–F*, and 9*B*, the time course of each phenomenon after skin injury is schematically indicated as a *curve.* The peak of each phenomenon is indicated as 100%. *D*, scheme indicates our working hypothesis. *, *p* < 0.05; **, *p* < 0.01; ***, *p* < 0.001.

To elucidate the cell fate of binuclear (tetraploid) fibroblasts after experimental injury, we performed FISH analyses *in vivo*. As shown in Fig. 8*A*, almost all nuclei showed two FISH signals per chromosome in *VIM^WT/WT^* or *VIM^WT/SA^* fibroblasts. However, >2 spots of chromosome 12 and/or 19 existed in *VIM^SA/SA^* fibroblasts (Fig. 8*A*); such aneuploid fibroblasts were observed diffusely rather than locally (data not shown). The percentage of cells with such an aberrant chromosome number significantly increased at day 7, peaked at day 15, and decreased thereafter (Fig. 8*D*). The increase in aneuploid fibroblasts followed the decrease in binuclear fibroblasts with extra centrosomes (Figs. 8*D versus* 6, *E* and *F*; also see Fig. 9*C*). At day 9 after the injury, γ-H2AX, a DNA damage or replication stress marker (56–59), was significantly elevated in *VIM^SA/SA^* fibroblasts (Fig. 8, *B* and *E*). This nearly coincided with senescence-associated β-galactosidase expression in *VIM^SA/SA^* fibroblasts (Fig. 8, *C* and *F*). The percentage of senescence-associated β-galactosidase-positive fibroblasts peaked later than that of γ-H2AX-positive cells (Fig. 8, *E versus F*; also see Fig. 9*C*).

We next performed real time PCR analyses to check mRNA expression of p21 (a representative of p53-responsive genes), p16^INK4a^, and p19^ARF^ (senescence-related genes) (40–43). As shown in Fig. 9*A*, mRNA expression of these three genes was significantly elevated in *VIM^SA/SA^* fibroblasts after injury, compared with *VIM^WT/WT^* or *VIM^WT/SA^* fibroblasts. However, the pattern of elevation differed between p21 and p16^INK4a^/p19^ARF^ (Fig. 9, *A* and *B*). In *VIM^SA/SA^* mice, p21 elevation was biphasic (Fig. 9*B*). The first phase was observed from day 3 to 7 after the injury (Fig. 9*B*). This initial elevation was observed in a *VIM^SA/SA^*-specific manner (Fig. 9*A*) and detected just after tetraploidization (Fig. 9*B versus* 6*E*; also see Fig. 9*C*). The second phase started around day 9, peaked at day 15, and declined thereafter (Fig. 9*B*). Because the transcript level of p21 was indistinguishable between *VIM^SA/SA^* fibroblasts and their littermate controls by day 15 (Fig. 9*A*), the latter elevation in *VIM^SA/SA^* mice might be linked to the wound repair process rather than the emergence of tetraploid/aneuploid cells. Moreover, the transcript level of p16^INK4a^ and p19^Arf^ in *VIM^SA/SA^* fibroblasts started to increase at day 7, peaked at day 15, and declined thereafter (Fig. 9, *A* and *B*). Because this elevation pattern was similar to the emergence of SA-β-gal-positive fibroblasts, *VIM^SA/SA^*-specific elevation of p16^INK4a^/p19^ARF^ was likely associated with cellular senescence.

## Discussion

In this study, we have demonstrated that *VIM^SA/SA^* mice exhibit subcutaneous fat loss and impaired wound healing, features of progeria (40, 41, 44). Our model is summarized in Fig. 9*D*. The inability of vimentin to become phosphorylated during mitosis induces an IF-bridge connecting the two daughter cells immediately after the first round of mitosis. Cells connected by an IF-bridge can undergo two distinct decisions of cell fate. Disruption of the IF-bridge between two daughter cells, likely by cell adhesion-dependent traction force (60) ultimately results in completion of cytokinesis (33). Such a compensatory event likely limits the range of pathological phenotypes seen in *VIM^SA/SA^* mice. Alternatively, a failure to undergo cytokinesis results in the formation of tetraploid cells with two centrosomes (31). In *VIM^SA/SA^* mice, binucleated cells were detected in lens epithelial cells (36) and in subcutaneous fibroblasts during an acute phase of wound repair (this study). In both settings, vimentin expression is relatively high. In intact subcutaneous tissue where vimentin expression is lower compared with that before, the rate of binucleation or tetraploidy is less prominent. Thus, the amount of vimentin per cell is one of the critical factors for cytokinetic failure-induced tetraploidy in *VIM^SA/SA^* mice.

Just after tetraploidization, p21 (a p53-responsive gene) transcript is elevated in a *VIM^SA/SA^*-specific manner (Figs. 9*B versus* 6*E*; also see Fig. 9, *A* and *C*). Thus, tetraploidy checkpoint (16–19) likely functions in *VIM^SA/SA^* mice during wound repair process. However, our results also indicate that tetraploid fibroblasts induced by cytokinetic failure enter a new cell cycle because more than three centrosomes are detected in these cells (Fig. 6, *D* and *F*; also see Fig. 7, *I* and *J*). Why do tetraploid *VIM^SA/SA^* fibroblasts override this p53-dependent checkpoint? A recent report suggests that the tetraploidy checkpoint can be over-ridden under several cell culture conditions, such as higher serum concentrations (20). Thus, some of the tetraploid fibroblasts can bypass the checkpoint and then enter a new cell cycle. During the second round of mitosis, these tetraploid fibroblasts with extra centrosomes likely develop into aneuploid fibroblasts, based on the reduced number of binuclear fibroblasts coinciding with increased numbers of aneuploid fibroblasts during wound healing (Fig. 6, *E* and *F, versus* 8*D*; also see Fig. 9*C*). This model is supported by the previous observation that cell division with extra centrosomes often exhibits a significant increase in chromosome mis-segregation, including lagging chromosomes during anaphase (61). Why is γ-H2AX elevated after the emergence of aneuploid fibroblasts? One possible explanation is the accumulation of DNA damage in aneuploid cells because DNA breaks are frequently generated by mitotic errors in chromosome segregation (62, 63). The accumulation of DNA damage may elevate the expression of senescence-related genes by a mechanism similar to previous reports (64, 65). The other explanation is the possible existence of DNA replication stress in aneuploid cells because γ-H2AX is also elevated in response to replication stress (59). Interestingly, the replication stress itself can be one of aging drivers at least in hematopoietic stem cells (59). Finally, these senescent fibroblasts may result in the dysfunction of subcutaneous tissue.

Our *VIM^SA/SA^* mice represent the first tetraploidy-prone mouse model with accelerating aging phenotypes, such as lens cataract (36), subcutaneous fat loss, and impaired wound healing (this study). These phenotypes are very similar to those in aneuploidy-prone, *BubR1^H/H^* mice (44). However, unlike *BubR1^H/H^* mice (44), the phenotypes in *VIM^SA/SA^* mice are likely restricted to tissues in which vimentin is highly expressed. Hence, we observed no significant phenotypes in liver, kidney, heart, and aorta (Figs. 2 and 3). In addition, *VIM^SA/SA^* mice were alive at least for 2 years, like their littermates (data not shown). Interestingly, cardiovascular dysfunction is well correlated with a short life span in *BubR1^H/H^* mice (especially in male mice) (66, 67). *VIM^SA/SA^* mice may have a normal life span, likely due to no apparent disorders in the cardiovascular system (Fig. 3).

The subcutaneous fat layer increased at the age of 3 months in our binucleation-prone *VIM^SA/SA^* mice (Fig. 1), although no significant changes were reported at the age of 2 months in aneuploidy-prone *BubR1^H/H^* progeria mice (44). One possible explanation is the difference in the mode of mitotic failure. Cytokinetic failure accompanies doubled numbers not only of chromosomes but also of centrosomes, whereas BubR1 insufficiency mainly induces chromosome mis-segregation, resulting in aneuploidy (44). Rho GTPase activity is inhibited by the existence of doubled centrosomes in tetraploid cells originating from cytokinetic failure (20). This promotes differentiation of mesenchymal stem cells (MSCs) to adipocytes (20, 68). Interestingly, subcutaneous adipocytes appear to increase in ploidy likely due to cytokinetic failure (Fig. 1*G*). MSCs failing cytokinesis may preferentially differentiate into adipocytes in young *VIM^SA/SA^* mice. The delay in wound repair at 3 months of age (Fig. 4) may be caused by the abnormalities in subcutaneous tissue, such as the composition of cutaneous progenitor cells, adipocytes, and fibroblasts (Fig. 1). In addition, the defect in cytokinesis may also deplete MSCs, resulting in premature subcutaneous fat loss at a late age.

Vimentin knock-out (*VIM*^−/−^) mice were reported to exhibit a similar defect in wound repair (49). However, except for the above phenotype, *VIM*^−/−^ mice exhibited quite different phenotypes, such as lymphocyte or platelet dysfunction (69–72). These dissimilarities are due to the following difference. The knock-out mouse model reflects the complete absence of vimentin, whereas our mouse model reflects the disturbance in rearrangement of mitotic vimentin filaments due to compromised mitotic vimentin phosphorylation. With regard to lens cataract, a mutation at Glu-151 to Lys (an EK mutation) was also reported in the human *VIM* gene (73). The patients suffering the above congenital cataract showed a dominant inheritance pattern (73), whereas our mice exhibited a recessive phenotype because no phenotypes, including lens, were observed in *VIM^WT/SA^* mice. This is likely due to a different influence of the above mutations on vimentin filament networks. EK mutants were reported to form vimentin aggregates, which resulted in the disruption of endogenous vimentin network (73). However, outside mitosis (in interphase), the SA mutants behaved like WT vimentin not only in transfected cultured cells (34) but also in our mouse model (36). Therefore, the above vimentin deficiency or mutation partially shows symptom(s) similar to SA mutation on vimentin in mice, but the underlying pathologies are quite different from each other.

In conclusion, we have demonstrated that cytokinetic failure-induced tetraploidy triggers age-related processes in subcutaneous fibroblasts of *VIM^SA/SA^* mice. It is known that tetraploid cells with genetic alterations of cancer-related genes exhibit chromosomal instability and can promote carcinogenesis (9, 10). Which factor(s) determine the fate of tetraploid cells *in vivo*? We hypothesize that the cell type and mutation states are major determinants of tetraploid cell fate. Here, we present strong evidence that mesenchymal cells, including subcutaneous fibroblasts, are associated with premature aging rather than carcinogenesis. Previous studies mainly focused on epithelial cells (9, 10). With additional alteration(s) in cancer-related gene(s), *VIM^SA/SA^* mice might exhibit cancer-prone phenotypes. Our mouse model will enable us to dissect the cross-talk between genetic alterations of cancer-related genes and tetraploidy for aging and carcinogenesis.

## Footnotes

2 The abbreviations used are:

IF: intermediate filament
MSC: mesenchymal stem cell
VIM: vimentin
MEF: mouse embryonic fibroblast.
